# Towards a better understanding of the posttreatment hemodynamic behaviors in femoropopliteal arteries through personalized computational models based on OCT images

**DOI:** 10.1038/s41598-021-96030-2

**Published:** 2021-08-17

**Authors:** Can Gökgöl, Yasushi Ueki, Daniel Abler, Nicolas Diehm, Rolf P. Engelberger, Tatsuhiko Otsuka, Lorenz Räber, Philippe Büchler

**Affiliations:** 1 ARTORG Center for Biomedical Engineering Research, University of Bern, Freiburgstrasse 3, 3010 Bern, Switzerland; 2 Department of Cardiology, Inselspital, Bern University Hospital, University of Bern, Freiburgstrasse 18, 3010 Bern, Switzerland; 3 Clinical and Interventional Angiology, Vascular Institute Central Switzerland, Aarenaustrasse 2 b, 5000 Aarau, Switzerland; 4 Department of Angiology, HFR Freiburg - Kantonsspital, Chemin des Pensionnats, 1708 Freiburg, Switzerland

**Keywords:** Biomedical engineering, Peripheral vascular disease, Translational research, Computational science

## Abstract

The hemodynamic behavior following endovascular treatment of patients with peripheral arterial disease plays a significant role on the occurrence of restenosis in femoro-popliteal (FP) arteries. The atheroprone flow conditions that are generally accepted to promote restenosis can be calculated by computational fluid dynamics (CFD) analyses, and these results can be used to assess individualized treatment outcomes. However, the impact of endovascular therapy on the flow behaviors of FP arteries are still poorly understood, as the imaging modalities used in existing numerical works (X-ray angiography, computed tomography angiography) are unable to accurately represent the post-treatment arterial geometry due to their low resolutions. Therefore, this study proposes a new algorithm that combines intra-arterial lumen geometry obtained from high-resolution optical coherence tomography (OCT) images with centerlines generated from X-ray images to reconstruct the FP artery with an in-plane resolution of 10 µm. This superior accuracy allows modeling characteristic geometrical structures, such as angioplasty-induced arterial dissections, that are too small to be reconstructed with other imaging modalities. The framework is applied on the clinical data of patients treated either with only-percutaneous transluminal angioplasty (PTA) (n = 4) or PTA followed by stenting (n = 4). Based on the generated models, PTA was found to cause numerous arterial dissections, covering approximately 10% of the total surface area of the lumen, whereas no dissections were identified in the stented arteries. CFD simulations were performed to investigate the hemodynamic conditions before and after treatment. Regardless of the treatment method, the areas affected by low time-averaged wall shear stress (< 0.5 Pa) were significantly higher (*p* < 0.05) following endovascular therapy (pre-PTA: 0.95 ± 0.59 cm^2^; post-PTA: 2.10 ± 1.09cm^2^; post-stent: 3.10 ± 0.98 cm^2^). There were no statistical differences between the PTA and the stent groups. However, within the PTA group, adverse hemodynamics were mainly concentrated at regions created by arterial dissections, which may negatively impact the outcomes of a leave-nothing-behind strategy. These observations show that OCT-based numerical models have great potential to guide clinicians regarding the optimal treatment approach.

## Introduction

Atherosclerosis affecting the femoro-popliteal (FP) arteries is mainly treated by endovascular therapy^1–3^. However, post-treatment complications that lead to restenosis negatively impact the clinical mid-to long-term success of the initial treatment. These complications are more common for patients that undergo stent implantation than for patients treated only by Percutaneous Transluminal Angioplasty (PTA). Several clinical studies performed on cadavers and patients with Peripheral Arterial Disease (PAD) have shown that stents alter the leg flexion-induced physiological deformation patterns of the FP arteries and their presence in high deformation zones, such as the popliteal segment, may cause arterial kinking, and potentially lead to restenosis^4–10^. Furthermore, it was found that stents disrupt the natural hemodynamic conditions within the superficial femoral (SFA) and popliteal artery (PA), causing flow disturbances that are considered a key contributing factor to restenosis^5, 11–13^.

The problems that occur in the presence of stents have informed the clinicians’ approach to the treatment of PAD. As a result, performing only PTA, i.e. leaving no permanent scaffold behind, has started to gain more attraction among clinicians^14, 15^. A possible problem with this strategy is the arterial dissections, which are formed due to the mechanical forces the PTA balloon exerts onto the artery during the revascularization procedure. Without a stent to push them towards the artery wall, the dissected layers can form false lumens that may cause abnormalities in the blood flow, which may undermine the success of “leaving-nothing-behind”^16^. As such, understanding how these complex lumen geometries affect FP artery hemodynamics are crucial to objectively assess the performance of different treatment methods.

Computational fluid dynamics (CFD) analyses provide an important framework to quantify the atherosclerosis-prone and -protective hemodynamic conditions in the artery, and can help predict whether the selected treatment will fail in the long-term. However, there are only a few numerical studies that investigated the flow behaviors in FP arteries following endovascular therapy^11–13, 17–19^. Gökgöl et al. used a custom-made algorithm to reconstruct the three-dimensional post-treatment arterial geometries of 20 patients from multiple 2D X-ray images^11^. The study was the first to evaluate changes in the post-treatment flow conditions due to leg flexion and show a correlation between adverse hemodynamics and restenosis. However, due to the limitations of the X-ray imaging, the models could include neither the stent geometry nor the arterial dissections caused by the PTA balloons. Colombo et al. developed and validated an algorithm to construct stented arterial geometries directly from computational tomography (CT) images and analyzed different wall shear stress (WSS) descriptors of a single patient^19^. In a follow-up study, they applied this framework to the dataset of 10 patients, which underwent CT imaging 1-week, as well as 1-year, after Nitinol stent implantation to show a relationship between atheroprone flow conditions and lumen remodeling^12^. However, due to the low resolution of CT imaging, the models lack detailed description of the lumen surface and the stent geometry. Finally, Conti et al. have performed CT angiography (CTA) on a single patient at a one-year follow-up of a popliteal aneurysm repair through stent-graft implantation^18^. They used this data as the basis of a proof-of-concept study, in which they performed CFD simulations and investigated the flow behaviors in the artery for straight- and bent-leg positions. In a separate study, they extended this approach to the dataset of two patients and analyzed the influence of different flow models, as well as different boundary conditions, on the hemodynamic conditions^17^. However, as CTA is not routinely used during aneurysm repair or to track disease progression, the immediate post-treatment arterial geometry had to be assumed from the one-year outcome. Furthermore, the stent geometry was not modeled. In summary, all of the above-mentioned studies highlight the importance of using intra-vascular imaging methodologies to obtain more accurate patient-specific data.

The main objective of this study was to investigate FP artery hemodynamics following endovascular treatment through patient-specific CFD simulations. Taking into account the limitations of the previous studies, patient-specific arterial anatomies have been reconstructed from a combination of X-ray angiography and optical coherence tomography (OCT) images to generate the lumen interface with an in-plane resolution of 10 µm. As a result, the effects of the aforementioned consequences, such as arterial dissections, on the flow behavior of FP arteries could be investigated for the first time.

## Methods

### Clinical data acquisition

#### Ethical approval

This study was approved by the ethical review committee of the Ethikkommission Nordwest- und Zentralschweiz (EKNZ 2014-119). All procedures were performed in accordance with the Declaration of Helsinki. Informed consent was obtained from all individual participants included in the study.

#### Patient characteristics

Eight patients (mean age: 72 ± 6; two females), presented for workup of suspected PAD, underwent initial screening at the Vascular Institute Central Switzerland (Aarau, Switzerland) and HFR Fribourg (Fribourg, Switzerland). Based on duplex ultrasound (DUS) measurements, six patients were diagnosed with Fontaine stage IIb, while the remaining two were categorized as IIa. Arterial lesions were mainly located around the distal-SFA, with a mean lesion length of 61 mm ± 36 mm. The level of calcification, assessed on fluoroscopic images, ranged from moderate to severe, and three patients exhibited total occlusions. Also of note is that for one patient (P1), the region of interest (ROI) was located distal to another lesion, which was treated with a Nitinol stent during endovascular therapy.

The patients were scheduled for routine endovascular treatment and, following confirmation of eligibility, recruited into the study. Based on lesion characteristics and the response of the artery to initial balloon angioplasty, the clinicians opted to treat half of the patients with only PTA (2 × POBA; 2 × DCBs); while the rest were implanted with Nitinol stents (2 × drug-coated; 2 × non-coated). Detailed information on individual lesion characteristics and their corresponding treatments can be found in Table 1.

**Table 1:** Patient demographics, lesion characteristics, treatment methods, clinical outcomes at a 1-year follow-up, and the time points, when OCT and DUS were performed.

		P1	P2	P3	P4	P5	P6	P7	P8
**Baseline**									
Age (in years)		76	82	71	63	74	65	73	73
Gender (Male/Female)		F	F	M	M	M	M	M	M
Fontaine stage (I–IV)		IIb	IIb	IIa	IIb	IIb	IIa	IIb	IIb
Lesion location		Distal SFA	Popliteal PII	Distal SFA/Popliteal P1	Distal SFA	Mid-/Distal SFA	Mid-/Distal SFA	Distal SFA	Mid- / Distal SFA
Lesion length (mm)		30	30	70	20	100	120	50	70
Occlusion (y/n)		n	n	n	n	y	y	y	n
Calcification level		Severe	Moderate	Moderate–severe	Moderate	Severe	Moderate	Severe	Moderate – Severe
**Treatment**									
PTA (device brand) diameter × length (mm)		Pirouette 35 5 × 40	Pirouette 35 5 × 40	Lutonix 35 6 × 120	InPact Admiral 35 5 × 60	Pirouette 35 6 × 40	InPact Admiral 35 6 × 60	Freeway 35 5 × 120	Passeo-18 Lux 5 × 80
Stent (device brand) diameter × length (mm)		–	–	–	–	Zilver PTX 6 × 120	Zilver PTX 6 × 150	Pulsar-18 7 × 60	Pulsar-18 7 × 40
**Follow-up**									
Fontaine stage (I–IV)		I	I	I	I	I	I	I	I
ABI		1	0.8	1.3	0.93	1	-	0.98	1.09
Binary restenosis (y/n)		n	n	n	n	n	n	n	n
TLR (y/n)		n	n	n	n	n	n	n	n
**Data acquisition**									
OCT	Pre-PTA	x	x	x	x				
	Post-PTA	x	x	x	x	x	x		x
	Post-Stent					x	x	x	x

*SFA* superficial femoral artery, *PTA* percutaneous transluminal angioplasty, *ABI* Ankle-Brachial Index, *OCT* optical coherence tomography, *DUS* Duplex ultrasound, y/n represents yes/no conditions.

A clinical follow-up performed at 12-months collected information regarding the Fontaine stage, ankle-brachial index (ABI), binary restenosis and TLR rates through DUS measurements, as well as a walking-impairment-questionnaire (Table 1). None of the patients exhibited clinical deterioration in lower limb perfusion. As such, no additional clinical imaging (i.e. X-ray, OCT) was performed during follow-up.

#### X-ray angiography

Prior to the start of the treatment, a calibration phantom with radiopaque fiducials was attached to the patients’ diseased legs. As part of the diagnostic angiography, two orthogonal X-ray images, separated by an angle of > 45°, were acquired at the ROI. Care was taken that the majority of the fiducials were visible in the images. The acquisition was performed at every stage of the treatment (pre-PTA, post-PTA, and, when applicable, post-stent) with the treated leg in supine position. All acquisitions were performed in cine mode to obtain high quality images. More information on the calibration phantom can be found in Schumann et al.^20^, while its utilization in FP arteries can be found in Schumann et al.^21^ and Gökgöl et al.^4^.

#### Optical coherence tomography

While the use of intra-arterial imaging during endovascular treatment is recommended by the latest Percutaneous Coronary Intervention guidelines^22^, OCT acquisition in the FP arterial tract remains outside of the clinical routine. However, no significant complications regarding its use in the peripheral arteries have been reported.

OCT imaging was performed using the ILUMIEN OPTIS console (Light Lab, Abbott Vascular Inc, Santa Clara, CA, USA). All pullbacks were acquired under anticoagulation with heparin. A 2.7 French (Fr) C7 Dragonfly Optis imaging catheter (Light Lab, Abbott Vascular Inc, Santa Clara, CA, USA) was positioned distal to the ROI and motorized pullback OCT imaging was performed at a speed of 36 mm/s for a total length of 75 mm. During pullback, an automated injector pump (Bayer/Siemens Helthineers, Switzerland) was used to introduce 30 ml non-diluted contrast medium with an injection rate of 5.0 ml/s. Following each acquisition, the image quality was immediately checked on the console and the acquisition was repeated if necessary. The position of the imaging catheter and probe were recorded with a ruler and filmed via radiographic means.

Apart from repeat acquisitions performed due to quality concerns, the number of OCT pullbacks was limited to one per patient to reduce excessive contrast medium injection. Similar to the angiography protocol, the pullbacks were obtained with the leg in supine position. For patients that underwent only PTA, OCT was performed during pre- and post-PTA stages; whereas for subjects in which a stent was implanted, OCT images were acquired only at post-PTA and post-stent time-points. For P7, the OCT images were only acquired following stent implantation. The availability of the datasets for each patient is reported in Table 1.

### Reconstruction of the arterial models

Two main components are required to generate an accurate reconstruction of the arterial geometries using the clinical data obtained during endovascular treatment; the arterial centerline, which represents the native tortuosity of the artery, and the arterial lumen.

#### 3D Arterial centerline generation

Utilizing a previously developed reconstruction algorithm, ArtRex (ARTORG Center for Biomedical Engineering Research, University of Bern, Bern, Switzerland), the 3D arterial centerlines of the ROI were obtained from X-ray angiographic images^4, 21^. As the details of this algorithm has already been discussed in multiple studies, only a brief overview will be provided here.

The complete angiographic dataset was saved in Dicom format. The two views taken at the ROI were selected and calibrated using the fiducials of the phantom; the fiducials were selected in a specific order and the calibration was automatically performed based on their voxel coordinates. The calibrated images were, then, imported into ArtRex, where the first step was to identify the distal- and proximal-ends of the ROI based on the initial and final positions of the OCT imaging catheter, respectively. Additional landmarks defined on OCT images, such as side-branches, also helped with this procedure. Once the ROI was well-established, the boundaries of the arterial lumen in both images were segmented using b-splines. Finally, the 2D boundaries were triangulated into 3D space using the calibration information and the 3D arterial centerline was computed from the 3D boundary lines.

#### Arterial lumen generation

The OCT image dataset was exported from the OCT console in native (raw) format and transferred to the Core Laboratory (CoreLab) at the Inselspital (University Hospital Bern, Bern, Switzerland). The pullbacks were imported into the offline review workstation, where they were first analyzed for quality and compared with the X-ray images to pinpoint the slices that corresponded to the ROI. Using the dedicated image analysis software, QCU-CMS (LKEB, Leiden University, Leiden, The Netherlands), the ROI was assessed every 2 slices (corresponding to an interval of 0.4 mm). The lumen contour, including the arterial dissections, was drawn as b-splines by the semi-automated detection feature of QCU-CMS and manual corrections were performed wherever required. Frames were excluded when more than one quadrant of the lumen border was not visible due to insufficient blood clearing or being out of frame.

The images with the embedded spline information were saved as .bmp files and transferred to an image segmentation software, Amira v5.4.5 (Thermo Fisher Scientific, Waltham, MA, USA), in which the lumen was defined as the area inside the splines through a threshold-based segmentation approach. A basic interpolation was made to extend the segmentation mask to the slices, where the lumen was not outlined with QCU-CMS. The resulting label image represented the arterial lumen on a straight line with a voxel size of 0.0102 × 0.0102 × 0.2 mm.

#### Mapping the OCT-based arterial lumen onto the X-ray arterial centerline

The final stage in obtaining the patient-specific arterial geometry was to warp the label image containing the straight arterial lumen onto the arterial centerline using an in-house framework developed in Python 3 and Matlab R2011b (The Mathworks Inc, Natick, MA, USA). First, the label image derived from OCT was down-sampled to an isotropic resolution of 0.075 × 0.075 × 0.075 mm^3^ for computational efficiency. This was followed by extracting the coordinates and values of each voxel pertaining to the lumen to construct an OCT-based 3D arterial centerline, which was, then, rigidly aligned with the 3D patient-specific centerline obtained from the X-ray images.

A dense displacement field was calculated to map each voxel of the resampled OCT image into its warped configuration. This forward displacement field was calculated as follows; the parametric position of each image slice along the OCT centerline was determined on the X-ray centerline based on arterial side branches. A rigid transformation was performed to position the image slices at the target location, which was followed by a rigid rotation to make each slice orthogonal to the X-ray centerline. Using this approach, a displacement vector was calculated for each voxel of the lumen identified from the OCT image.

**Figure 1:**
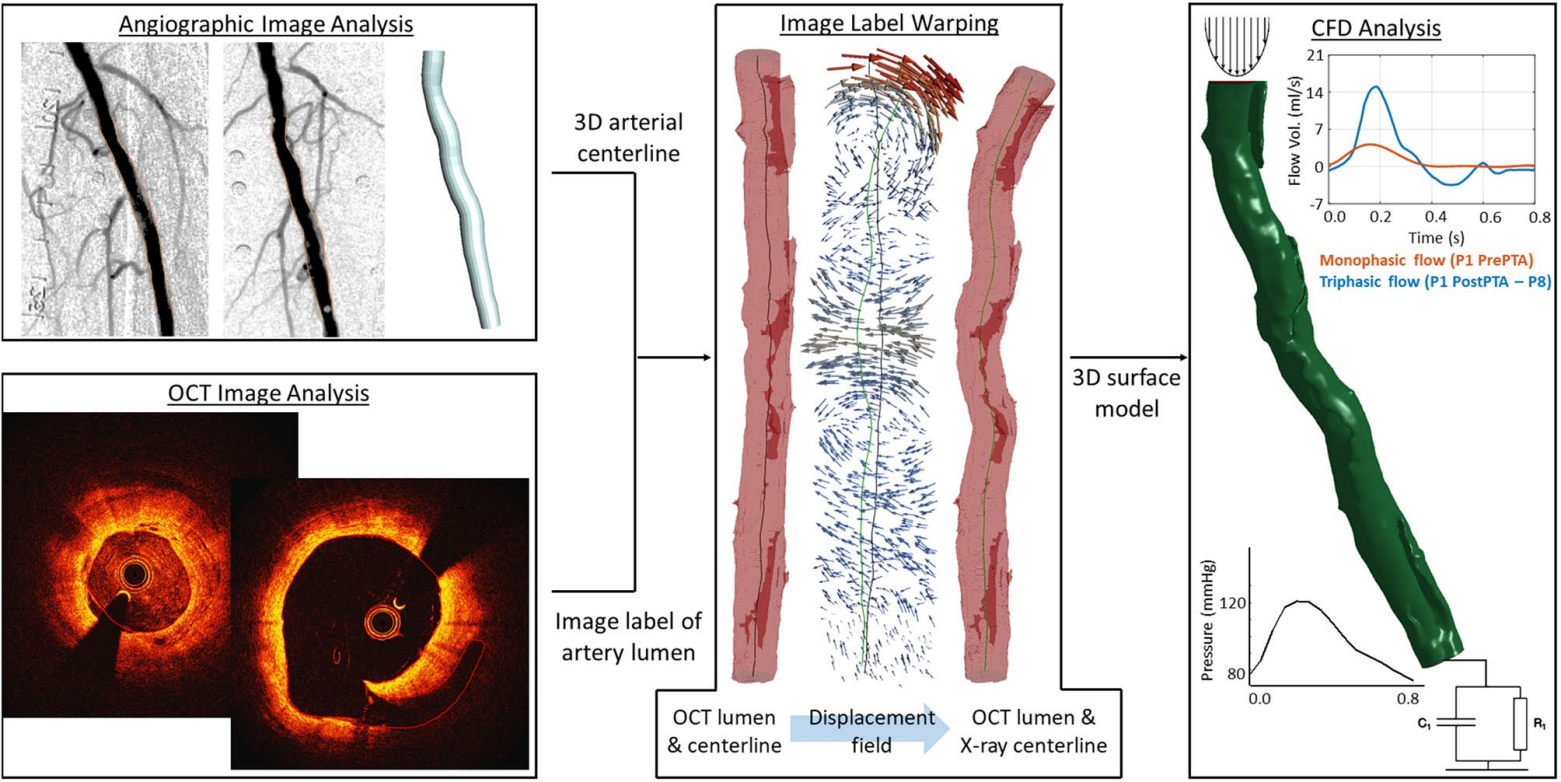
Outline of the reconstruction framework to generate 3D models of the artery from clinical data and obtain flow markers related with restenosis: The arterial centerline is generated from two orthogonal angiographic images, where the boundary of the artery lumen is identified using b-splines (in yellow) and triangulated into 3D-space (top-left); the lumen geometry, including arterial dissections, is segmented by b-splines (in red) from OCT images with sub-millimeter accuracy (bottom left); the image label containing the OCT-based lumen geometry in voxel format is warped onto the X-ray arterial centerline (in green) based on the displacement field computed between the OCT (in black) and X-ray centerlines (middle); and, finally, the surface model generated from the warped image label is meshed and transferred to a CFD solver, where a parabolic flow profile and a two-parameter Windkessel model are applied to the inlet and outlet, respectively. (Generated by LS-DYNA R10.1, http://www.lstc.com/products/ls-dyna).

From this deformation field, the dimensions of the warped, target image was identified. An empty target image with the same isotropic resolution of 0.075 mm^3^ was generated by extending the domain of the original OCT label image to cover these dimensions. The final warping step was performed by, first, computing a dense inverse deformation field using ‘SimpleITK’, which provided the displacement field that transformed each voxel of the target image into their corresponding position in the original OCT dataset. This deformation field was, then, used to define the label map of the warped OCT image in the target image space using the ‘Advanced Normalization Tools’ (ANTS). Figure 1 illustrates the warping of the OCT lumen to the arterial centerline obtained from X-ray imaging.

#### Surface mesh generation

The warped label image was transferred back to Amira and, after performing a threshold-based segmentation, an initial surface model of the patient-specific arterial lumen was generated with triangular elements. To smooth reconstruction irregularities, the triangulated surface mesh was regularized using the Laplacian smoothing filter (MeshLab v2016.12). Finally, both ends of the model were clipped (Meshmixer v3.5, Autodesk Inc., San Rafael, CA, USA) to provide distinct boundaries to apply inlet and outlet boundary conditions in the CFD solver. In addition, both ends were slightly extended to avoid boundary effects. More information regarding the geometrical properties and meshes for each model can be found in Table 2.

**Table 2:** The geometrical and FE mesh properties of the patient-specific arterial models reconstructed from OCT and X-ray images.

Patients	Time points	Inlet location	Length (mm)	Inlet area (mm^2^)	Total Area (cm^2^)	# of surface elements	# of volume elements
P1	Pre-PTA	Distal to upstream lesion	49.03	10.76	4.44	197,988	5,328,000
	Post-PTA	Distal to upstream stent	50.53	14.30	7.97	236,736	7,111,860
P2	Pre-PTA	Proximal to lesion	56.25	13.39	6.11	206,240	5,647,489
	Post-PTA	Proximal to lesion	56.03	12.82	9.02	222,788	6,371,831
P3	Pre-PTA	Proximal to lesion	63.33	10.81	6.53	225,686	6,255,287
	Post-PTA	Proximal to lesion	62.64	16.19	9.85	221,462	6,179,122
P4	Pre-PTA	Proximal to lesion	68.02	17.92	10.94	232,894	6,616,151
	Post-PTA	Proximal to lesion	68.81	16.79	11.34	228,434	6,450,666
P5	Post-PTA	Intra-lesion	60.35	9.45	7.75	222,052	6,120,032
	Post-Stent	Intra-stent	60.52	14.82	10.33	229,954	6,372,419
P6	Post-PTA	Intra-lesion	62.88	16.73	8.32	260,382	7,215,475
	Post-Stent	Intra-stent	61.64	20.23	9.47	265,200	7,510,878
P7	Post-Stent Str	Intra-stent	54.90	24.82	9.99	254,698	7,524,110
	Post-Stent Flx	Intra-stent	54.90	25.57	9.67	275,128	7,956,485
P8	Post-PTA	Intra-lesion	52.47	15.2	7.70	278,216	8,199,703
	Post-Stent	Intra-stent	52.60	46.34	11.87	292,484	9,283,600

### CFD analyses

The smoothed and clipped surface mesh was imported as an .stl into the pre-processor of LS-DYNA (LST, ANSYS Inc., Canonsburg, PA, USA), where the CFD domain was defined by separating the surface model into three regions; the inlet, the outlet and the wall. The volumetric mesh of the domain was generated by utilizing the automatic mesh generation feature of the CFD solver of LS-DYNA, which uses a mesh growth scale factor to extend the surface elements that define the domain’s boundaries. Additionally, a boundary layer was introduced to inflate the initial mesh with additional tetrahedral elements and increase the accuracy of the flow variables at the boundaries. The scale factor and the properties of the boundary layer were chosen by evaluating specific flow parameters, such as the mean and maximum values of velocity and WSS, at the locations with minimum and maximum lumen diameter, as well as within the false lumens generated due to arterial dissections. The selected parameters for the generation of the volume mesh were deemed appropriate when the maximal differences between subsequent refinements were less than 4%, and that the number of iterations for the convergence of a time-step was between 4 and 6^23–25^. Based on this criterion, a scale factor of 1.8, and a boundary layer with 6 elements and a thickness of 0.15 mm were selected for all models to create the tetrahedral volumetric meshes.

The transient analyses were solved using LS-DYNA R10.1. A constant time-step of 2 × 10^–3^ was assigned for the fluid problem, as this value was found to satisfy fine convergence criteria. The flow was assumed to be laminar with blood being modeled as a Newtonian fluid (ρ = 1050 kg/m^3^; μ = 3.5 × 10–3 Pa s). Three cardiac cycles were simulated and only the variables computed for the last cycle were taken into consideration.

#### Boundary conditions

The boundary conditions were derived from DUS measurements of 24 patients obtained from a previous clinical study on FP arteries^4^. The average values for peak systolic velocity (PSV) and the minimum diastolic velocity (MDV) were computed for three arterial segments; proximal to lesion/treated region, within lesion/treated region, and distal to lesion/treated region (Table 3). Based on the inlet and lesion locations of the reconstructed models, two specific flow profiles were adopted from the work of Mohajer et al.^26^ (Fig. 1). For only one case (P1 Pre-PTA), who had its inlet located downstream to another lesion, the flow profile was assumed to be monophasic, whereas, for the rest of the cases, including the post-PTA configuration of P1, the flow was assumed to be triphasic. The volumetric flow rates (ml/s) were converted to velocity waveforms (cm/s) using the inlet areas and DUS values that corresponded to the inlet locations for each of the eight patients. The imposed velocity was applied at the inlet according to a parabolic distribution.

**Table 3:** The average velocities of 24 patients measured by DUS prior to and following endovascular treatment.

	Pre-PTA			Post-PTA			Post-Stent		
Velocity (cm/s) (Mean ± SD)	Prox. to lesion	Intra-lesion*	Distal to lesion*	Prox. to lesion	Intra-lesion	Distal to lesion	Prox. to stent	Intra-stent	Distal to stent
Peak Systole	88.30 ± 28.40	262.68 ± 77.32	43.01 ± 16.87	103.00 ± 48.21	81.60 ± 38.53	62.75 ± 24.36	123 ± 53.78	70.64 ± 33.87	60.63 ± 37.18
Min. Diastole	− 14.90 ± 3.42	–	–	− 17.20 ± 5.54	− 13.80 ± 3.09	− 13.67 ± 2.77	− 21.21 ± 4.63	−11.90 ± 4.51	−10.17 ± 1.25

*Represents monophasic flow.

A two-parameter Windkessel model, with a single resistance (R) and capacitance (C), was assigned at the outlet. For each patient, the parameters for the R and C were obtained based on the mass flow rate calculated at the inlet and an idealized pressure profile, which had systolic and diastolic pressure values of 120 mmHg and 80 mmHg, respectively (Table 4). A no-slip condition was prescribed at the artery wall and an initial pressure of 80 mmHg was applied to the entire domain.

**Table 4:** Parameters for the two-element Windkessel model.

Windkessel Parameters	P1		P2		P3		P4		P5		P6		P7		P8	
	Pre-PTA	Post-PTA	Pre-PTA	Post-PTA	Pre-PTA	Post-PTA	Pre-PTA	Post-PTA	Post-PTA	Post-Stent	Post-PTA	Post-Stent	Post-Stent Str	Post-Stent Flx	Post-PTA	Post-Stent
Resistance (MPa s/mm^3^)	4.0e−5	1.9e−5	1.3e−5	1.2e−5	1.6e−5	9.2e−6	9.6e−6	8.8e−6	2.0e−5	1.6e−5	1.2e−5	1.1e−5	7.7e−6	8.7e−6	1.2e−5	4.7e−6
Capacitor (mm^3^/MPa)	3.9e+4	8.6e+4	1.2e+5	1.3e+5	1.0e+5	1.7e+5	1.6e+5	1.8e+5	8.1e+4	1.0e+5	1.4e+5	1.5e+5	2.1e+5	1.8e+5	1.3e+5	3.4e+5

### Post-processing

**Figure 2:**
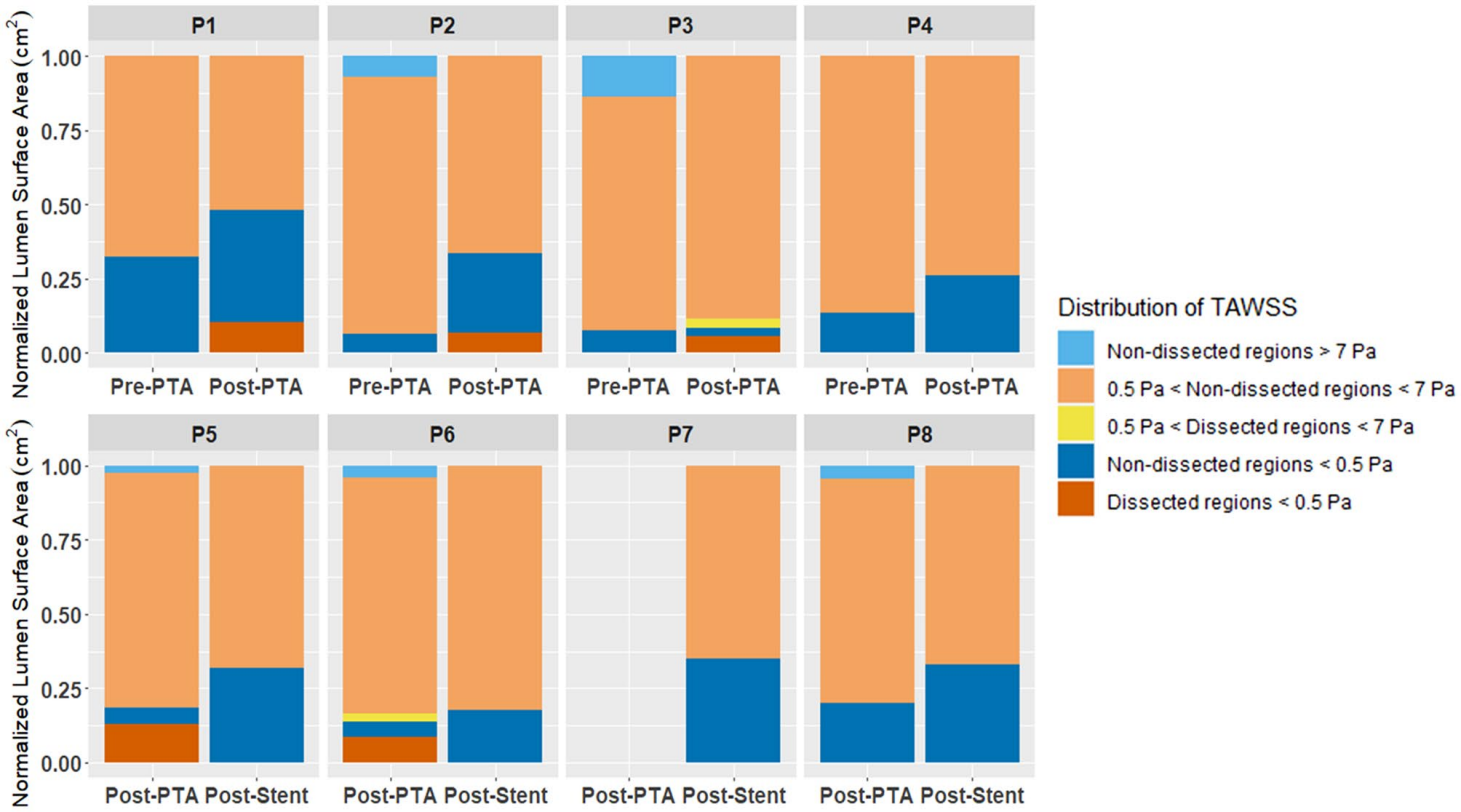
The normalized distribution of adverse hemodynamics (TAWSS < 0.5 Pa; TAWSS > 7 Pa) and normal flow behavior at the lumen surface for different segments of the artery. (Generated by R 3.5.0, https://www.r-project.org).

Based on the reconstructed models, the total surface areas of the arteries, as well as the surface areas of the false lumens generated by intimal tears, were evaluated to estimate the effects of endovascular treatment on the lumen geometries (Table 2). Time-averaged flow rates computed at the inlets revealed the differences between the boundary conditions adopted for each patient (Table 5). The hemodynamic behaviors have been analyzed as the areas affected by Time-averaged Wall Shear Stress (TAWSS), with adverse flow conditions being established as low TAWSS (< 0.5 Pa)^23, 27^ and high TAWSS (> 7 Pa)^28^ (Fig. 2). Finally, non-paired t-tests have been performed between pre-PTA, post-PTA and post-stent datasets to investigate statistical differences (*p* value < 0.05) between the different treatment time-points.

**Table 5:** The time-averaged flow rate values computed at the inlet for each patient.

	P1		P2		P3		P4		P5		P6		P7		P8	
	Pre-PTA	Post-PTA	Pre-PTA	Post-PTA	Pre-PTA	Post-PTA	Pre-PTA	Post-PTA	Post-PTA	Post-Stent	Post-PTA	Post-Stent	Post-Stent Str	Post-Stent Flx	Post-PTA	Post-Stent
Flow rate (ml/min)	23.72	52.05	72.24	81.13	61.13	104.81	99.85	108.94	49.06	61.68	83.21	88.04	124.21	111.06	77.80	163.53

## Results

Regardless of the ROI or any of the patient characteristics, endovascular treatment has led to an increase in the total surface area of the arterial lumen (Table 2). The average surface areas for pre-PTA, post-PTA and post-stent groups were estimated to be 7.00 cm^2^ ± 2.77 cm^2^, 8.85 cm^2^ ± 1.34 cm^2^ and 10.41 cm^2^ ± 1.03 cm^2^, respectively. With an increase of 20.83%, balloon angioplasty resulted in a slightly larger surface increase than Nitinol stent implantation, which produced only an additional 15% lumen gain following PTA.

A major difference between the effects of the two treatment methods on the lumen geometry was the severity of the arterial dissections. The OCT images obtained after PTA showed significant damage at the lumen interface in the form of intimal tears, which created several false lumens along the length of the treated segments. In total, five of the seven patients exhibited dissections after ballooning, while no visible tears were identified for patients P4 and P8. The average surface area of the false lumens was found to be 0.86 cm^2^ ± 0.15 cm^2^, which represents approximately 10% (± 2.39%) of the total surface area. In contrast, no dissections were observed within the stented segments; however, regions proximal or distal to the stent could still exhibit tears.

The time-averaged flow rate computed at the inlet for the pre-PTA group was 77.75 ml/min ± 31.53 ml/min, which is in line with the values observed in literature for patients with varying degrees of PAD (Table 5)^26^. The corresponding post-PTA group (P1-4) yielded an average flow rate of 86.73 ml/min ± 26.16 ml/min; whereas, the average post-PTA flow rate prior to stenting (P5-6 & P8) was 70.03 ml/min ± 18.35 ml/min. Following stent implantation, this value was recorded to be 109.71 ml/min ± 38.36 ml/min. Within each of the treatment groups, the average flow rate increased with each step of the endovascular therapy and this can be jointly attributed to the increases in the flow velocity and the inlet surface area. Among all the patients, P1 is noteworthy for having the lowest flow rate as its ROI was located below another flow-limiting lesion.

**Figure 3:**
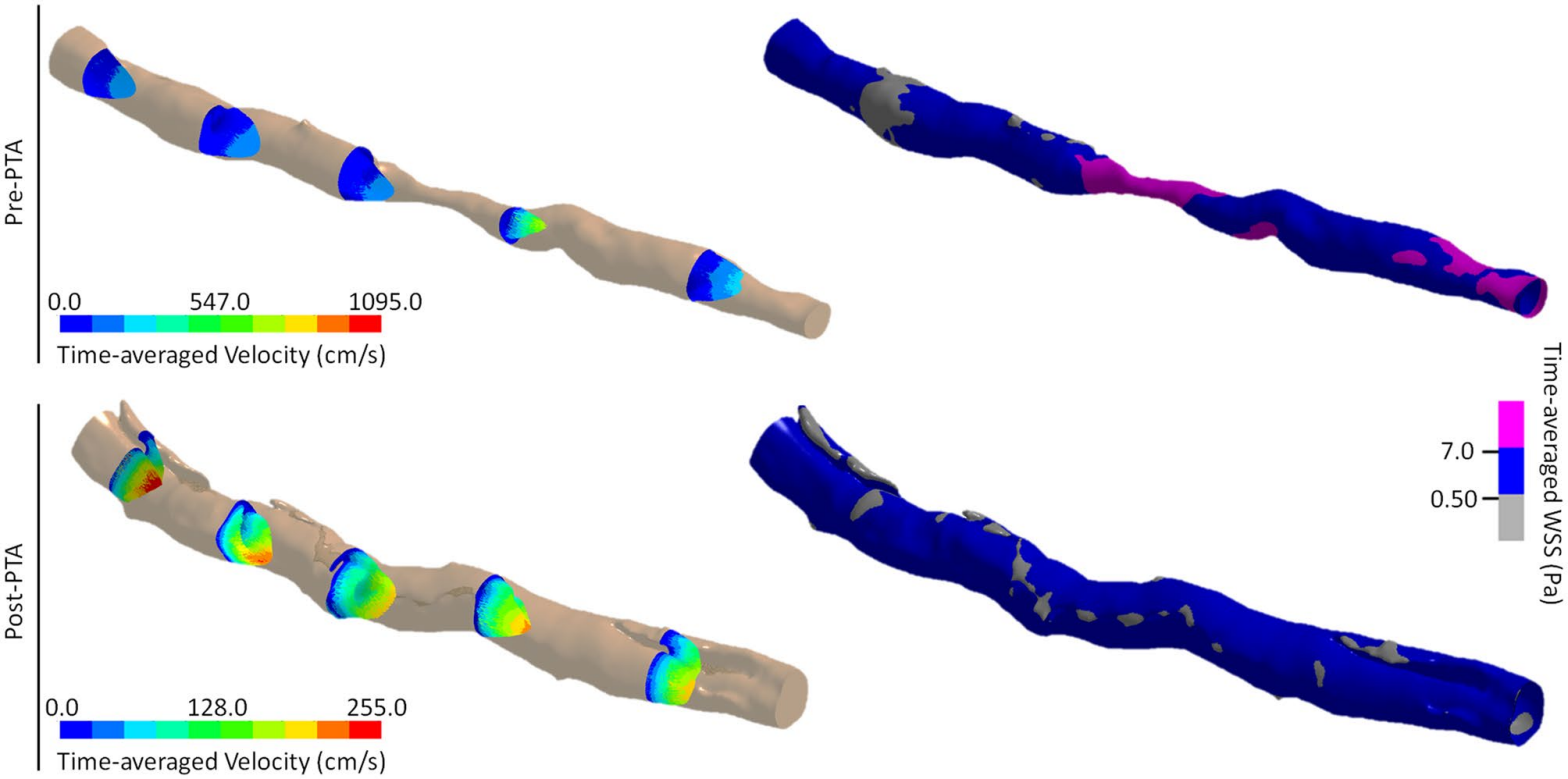
The development of time-averaged flow velocity and distribution of time-averaged Wall Shear Stress (TAWSS) in an artery (P3) before and after PTA. Prior to treatment, the maximum velocity is located at the lumen narrowing and the areas affected by low TAWSS are only located proximal to this section. The presence of low (grey) and high (magenta) TAWSS at this time-point is mostly related with localized diameter changes and skewness of the velocity profile, which occurs due to the natural tortuosity of the artery. Following PTA, there is a significant increase in the lumen diameter and multiple dissections can be observed along the length of the artery. The low velocity at these regions directly affects the WSS distribution, which shows that the areas affected by low TAWSS (grey) are mainly localized at the surfaces generated by dissections. (Generated by LS-DYNA R10.1, http://www.lstc.com/products/ls-dyna).

**Figure 4:**
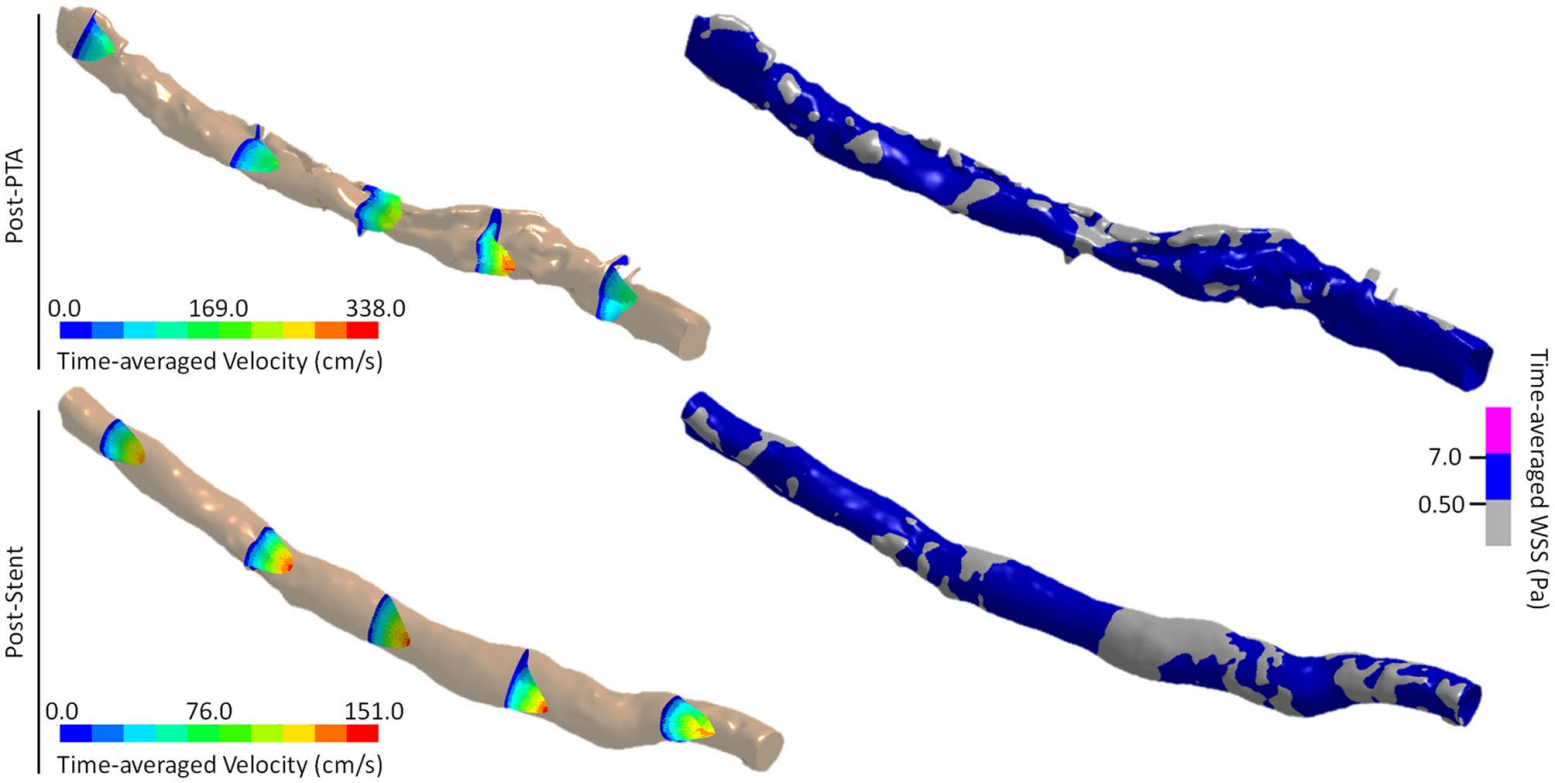
The development of time-averaged flow velocity and distribution of time-averaged Wall Shear Stress (WSS) in an artery (P5) following PTA and Nitinol stent implantation. As is the case with P3, the majority of the areas affected by low TAWSS is concentrated at arterial dissections, where the flow velocity is significantly lower than the rest of the artery. Following stenting, the dissected tissues are pushed towards the lumen wall and the symmetric velocity profile is mostly conserved along the artery’s length. At this time-point, the areas affected by low TAWSS are caused by localized deformities made by stent struts, as well as due to the distortions in the velocity profile affected by diameter changes and the tortuosity of the artery. (Generated by LS-DYNA R10.1, http://www.lstc.com/products/ls-dyna).

Prior to endovascular therapy, the mean area affected by low TAWSS was found to be 0.95 cm^2^ ± 0.59 cm^2^ (Fig. 2). Balloon angioplasty was found to significantly increase this baseline value, as the affected areas following PTA were computed to be 2.10 cm^2^ ± 1.09 cm^2^ (*p* = 0.048). In arteries with dissections, 87.75% (± 17.06%) of the surfaces covering the false lumens exhibited low TAWSS and, for three patients, 65% (± 6.08%) of the affected areas was concentrated at these locations (Figs. 3, 4). Similarly, the areas affected by low TAWSS in stented arteries were significantly higher than the pre-PTA configuration (3.10 cm^2^ ± 0.98 cm^2^, *p* = 0.013) (Fig. 2). Compared to the results of the PTA, the affected areas were not localized to certain regions, but more homogenously distributed along the length of the stented segments (Fig. 4). There were no statistically significant differences between the two treatment methods.

Compared to low TAWSS, high TAWSS at the artery walls was less present on the arterial surface. For the pre-PTA group, only two patients were affected by high TAWSS with a mean area of 0.33 cm^2^ ± 0.43 cm^2^ (Fig. 2). Following angioplasty, this value reduced to 0.11 cm^2^ ± 0.17 cm^2^, and after stenting, none of the arteries were affected by high TAWSS. Prior to treatment, the affected areas were mostly localized to regions with significant lumen narrowing due to the presence of stenosis. This observation extended to the post-PTA configuration, as the affected areas were present at segments, where the angioplasty could not achieve sufficient revascularization of the lumen. However, none of these differences were statistically significant.

## Discussion

The occurrence of restenosis following endovascular therapy of patients suffering from PAD continues to undermine the short and long-term outcomes of the treatment^29^. As shown by recent clinical and numerical studies, the choice of treatment method has a significant effect on the mechanical and hemodynamic behaviors of FP arteries and an incorrect assessment by the clinician may generate adverse conditions that promote restenosis^4, 11, 12^. However, existing research is limited by the use of imaging methodologies^12, 17–19^, which cannot accurately represent the arterial geometry, and, therefore, the results fail to provide conclusive evidence regarding the optimal treatment approach. As such, the main objective of this study was to develop a framework to build personalized models of FP arteries based on intra-arterial imaging. Reconstructed from an in-plane resolution of 10 µm, the OCT-based models will include a more detailed representation of the lumen interface. This will provide the possibility to investigate the effects of complex geometrical abnormalities that occur following endovascular therapy (i.e. intimal tears, stent strut imprints) on the hemodynamic behaviors of the artery.

The proper representation of the lumen surface within a numerical framework is crucial, as recent clinical investigations have reported that more than 80% the PAD patients treated only with balloon angioplasty had varying degrees of arterial dissections^15^. We observed a similar ratio among patients included in this study; the majority of the arteries exhibited dissections following PTA, which formed false lumens that made up approximately 10% of the total lumen surface. More importantly, the CFD analyses showed that the presence of dissections contributed to the adverse flow behaviors in the artery, as nearly all the surfaces covering these false lumens were affected by low TAWSS (Fig. 2). Apart from the known risk factors associated with intimal tears, the low flow velocity in these regions may lead to restenosis and completely obstruct the true lumen.

Compared to the pre-PTA configuration, both treatment methods had a statistically significant increase in the areas affected by low TAWSS. For the post-PTA group, the majority of the affected areas were concentrated within regions formed by dissections (Figs. 3, 4). However, for some patients, the adverse flow conditions extended beyond the false lumens and could be observed throughout the entire artery. While some of this is still related with the poor hemodynamic behaviors occurring within the immediate vicinity of the dissections, the rest can be attributed to a constantly changing lumen diameter along the length of the treated segment, as well as to the native tortuosity of the artery. During stent expansion, the integrity of the lumen was, somewhat, recovered, as the dissected tissues were pushed towards the rest of the wall. In the absence of any arterial dissections, the areas affected by low TAWSS could be seen at the localized deformities that stent struts made on the lumen surface. Similar to the post-PTA group, a number of significant diameter variations along the stented segment, as well as the curvature of the artery, also contributed to a further increase in the affected areas (Fig. 4).

It is very difficult to compare the current results with those of previous studies due to differences in clinical data, image acquisition method, geometrical properties of the models and the boundary conditions. Among the handful of studies that used patient-specific data to investigate post-treatment hemodynamics of the FP arteries, only Gökgöl et al. has analyzed the adverse flow conditions following PTA^11^. Based on the clinical data of 10 patients, the mean area affected by low TAWSS corresponded to 10% (± 10%) of the total lumen surface, which is lower than the mean percentage obtained from the current study (24% ± 13%). As the surfaces surrounding the false lumens accounted for approximately half of the affected areas, the difference can be attributed to the lack of dissections in the X-ray models, which may have led to a significant underestimation of flow behaviors related with low TAWSS. Colombo et al. has performed CFD simulations based on the CTA data of a single patient that underwent stent implantation^19^. As the surface area of the stented segment (11.51 cm^2^) was very similar to the average surface area of our post-stent group (10.41 cm^2^ ± 1.03 cm^2^), the percentage of adverse flow areas could directly be compared. With a threshold of 0.4 Pa, they estimated that 26% of the stented wall was affected by low TAWSS. Adapting our threshold to theirs, we found that only 14% ± 5% of the surface area had poor hemodynamics. While this difference can be attributed to a variety of modeling parameters (i.e. reconstruction method, boundary conditions), the results from a single patient is not enough to make an accurate comparison between the two frameworks.

Several works have used OCT to generate patient-specific models of arterial geometries^30–33^; however, these were mostly proof-of-concept studies and they focused, exclusively, on coronary arteries, where the utilization of intra-vascular imaging methods are more common. Although the use of OCT in the FP arterial tract has clinically proven to be feasible and safe^34, 35^, the existing research has mainly relied on simpler imaging methods, such as X-ray angiography, CTA or 3D rotational angiography, that do not allow the reconstruction of surface irregularities caused by balloon angioplasty^18, 36, 37^. To our knowledge, this is the first attempt to use OCT images in FP arteries as the basis of a numerical study.

The main idea behind previously developed semi-/automatic reconstruction algorithms used for coronary arteries was to, first, identify the lumen boundaries and, then, generate the lumen surface by lofting/blending the lumen contours. This approach works well as long as the integrity of the surface lumen remains intact, which is the case for pre-PTA and post-stent configurations. However, the extent of arterial dissections in FP arteries meant that this method would be unfeasible to generate the post-PTA lumen surface. The workflow presented in this paper is unique in the sense that arterial models were directly built from image labels, which ensured the accurate reconstruction of discontinuous surfaces caused by intimal tears (Fig. 1). Additionally, the possibility to resample the images prior to surface generation allows the user the flexibility to change the level of detail they would like to include in the models.

As one of the hypothesis behind high restenosis rates in the FP arterial tract is leg flexion related disturbances to the structural and hemodynamic behaviors of the artery, the success of the framework to reconstruct models in configurations other than the supine leg position was evaluated on a single patient. For P7, the data acquisition was also performed with the leg in a flexed position, which was established using a cast that ensured a knee/hip flexion of approximately 70°/20°. The algorithm generated the flexed model with the same accuracy as the straight models; however, it should be noted that, for the selected ROI, leg flexion did not cause any significant differences in the length, surface area or the curvature of the artery. Consequently, the areas affected by adverse flow conditions were similar for both straight and flexed artery models.

Several limitations of the current study need to be discussed. Although DUS was performed in all patients prior to and following treatment, there was only limited access to this data. This resulted in an incomplete representation of the flow behaviors for each patient. To remedy this, a population-specific approach was taken to derive an average inlet velocity for each treatment time-point based on the dataset of 24 patients with similar backgrounds and treatment histories. However, the lack of patient-specific boundary conditions may still impact the CFD results by introducing a mismatch between the flow velocity specified at the inlet and the geometrical properties of the artery models. While the number of patients included in this study is significantly higher than similar numerical works^18, 19^, more patients are required to make conclusive statements regarding the hemodynamics between different treatment groups. During follow-up, clinical image acquisition should be considered as this information can directly be used to validate the predictive capabilities of the numerical models. Additionally, care should be taken to reduce the variability among the clinical data, specifically the ROI, which is caused by the limitations of performing OCT in FP arteries. Typically, occlusions and heavy calcifications prevent the use of OCT prior to PTA, leading to missing datasets; and performing only a single pullback to limit the amount of contrast agent administered to the patient may result in an incomplete acquisition of the ROI. To mitigate this problem and ensure that the boundary conditions corresponded well with their respective ROIs, we adopted different velocity amplitudes and, for a single patient, profiles at the inlet. However, mixing different ROIs make the comparison between the patients difficult and introduces additional variability in the average results. Considering different arterial segments is not a problem when investigating the changes in the hemodynamics due to different treatment steps within each dataset, as the ROI before and after treatment is the same for each patient. However, the average results for the groups of pre-PTA and post-PTA should be interpreted carefully, as they represent different regions within the FP artery.

The identification of lumen contours from OCT images was based on a semi-automatic segmentation method followed by manual corrections. While this is considered to be the more robust method than semi-automatic method alone, it may have introduced operator errors to the reconstruction. This can be diminished by using a fully-automatic detection method or by including additional operators to reduce bias. The native resolution of the OCT images were down-sampled during the reconstruction of the 3D arterial geometries due to extensive computation time related with the warping of the OCT-based image labels onto the arterial centerlines. This means that some details of the lumen geometry may have been omitted from the final models. Nevertheless, the reconstruction accuracy used in this study was well below 0.1 mm. Finally, for stented arteries, stent geometry was not explicitly modeled. This may have had an effect on the flow behaviors and circulation zones within the ROI, as well as on the areas affected by adverse hemodynamics.

## Conclusion

This study presents a unique framework to build 3D arterial models from OCT and X-ray images and its applicability has been shown on the clinical data of eight patients with PAD that underwent different types of endovascular therapy. The algorithm is capable of reconstructing severe discontinuities in the surfaces caused by intimal tears, which occur following PTA. As a result, this was the first time that patient-specific models of FP arteries could include arterial dissections, and also the first time the hemodynamics in dissected FP arteries were investigated. While no significant differences were found in adverse flow conditions between Nitinol stent implantation and a PTA-only approach, the presence of false lumens was found to contribute to an increase in the surfaces affected by low TAWSS, which can compromise the decision to leave-nothing-behind. These observations show that the use of OCT-based numerical models has the potential to reveal the long-term effects of endovascular therapy in FP arteries and inform clinicians regarding the optimal treatment approach.

## Data Availability

The datasets generated during and/or analyzed during the current study are available from the corresponding author on reasonable request.
